# Hypertonic saline alleviates experimentally induced cerebral oedema through suppression of vascular endothelial growth factor and its receptor VEGFR2 expression in astrocytes

**DOI:** 10.1186/s12868-016-0299-y

**Published:** 2016-10-13

**Authors:** Linqiang Huang, Wei Cao, Yiyu Deng, Gaofeng Zhu, Yongli Han, Hongke Zeng

**Affiliations:** 1 Department of Emergency and Critical Care Medicine, Guangdong General Hospital, Guangdong Academy of Medical Sciences, Guangzhou, 510080 People’s Republic of China; 2 Zhuzhou Central Hospital, Zhuzhou, 412007 People’s Republic of China

**Keywords:** Hypertonic saline, Cerebral oedema, Vascular endothelial growth factor, Astrocyte, ZO-1, Claudin-5

## Abstract

**Background:**

Cerebral oedema is closely related to the permeability of blood–brain barrier, vascular endothelial growth factor (VEGF) and its receptor vascular endothelial growth factor receptor 2 (VEGFR2) all of which are important blood–brain barrier (BBB) permeability regulatory factors. Zonula occludens 1 (ZO-1) and claudin-5 are also the key components of BBB. Hypertonic saline is widely used to alleviate cerebral oedema. This study aimed to explore the possible mechanisms underlying hypertonic saline that ameliorates cerebral oedema effectively.

**Methods:**

Middle cerebral artery occlusion (MCAO) model in Sprague-Dawley (SD) rats and of oxygen–glucose deprivation model in primary astrocytes were used in this study. The brain water content (BWC) was used to assess the effect of 10 % HS on cerebral oedema. The assessment of Evans blue (EB) extravasation was performed to evaluate the protective effect of 10 % HS on blood–brain barrier. The quantification of VEGF, VEGFR2, ZO-1 and claudin-5 was used to illustrate the mechanism of 10 % HS ameliorating cerebral oedema.

**Results:**

BWC was analysed by wet-to-dry ratios in the ischemic hemisphere of SD rats; it was significantly decreased after 10 % HS treatment (*P* < 0.05). We also investigated the blood–brain barrier protective effect by 10 % HS which reduced EB extravasation effectively in the peri-ischemic brain tissue. In parallel to the above notably at 24 h following MCAO, mRNA and protein expression of VEGF and VEGFR2 in the peri-ischemic brain tissue was down-regulated after 10 % HS treatment (*P* < 0.05). Along with this, in vitro studies showed increased VEGF and VEGFR2 mRNA and protein expression in primary astrocytes under hypoxic condition (*P* < 0.05), but it was suppressed after HS treatment (*P* < 0.05). In addition, HS inhibited the down-regulation of ZO-1, claudin-5 effectively.

**Conclusions:**

The results suggest that 10 % HS could alleviate cerebral oedema possibly through reducing the ischemia induced BBB permeability as a consequence of inhibiting VEGF–VEGFR2-mediated down-regulation of ZO-1, claudin-5.

## Background

Cerebral oedema is an acute complication of various brain diseases such as cerebral ischemic stroke [1], and traumatic brain injury [2]. The development of cerebral oedema remains the most significant predictor of treatment outcome. If it is not treated timely and with an effective strategy, it could be life-threatening due to formation of cerebral hernia. To this end, osmotherapy is one of commonly used adjuvant therapies in clinical patients with cerebral oedema such as osmotic dehydrating agents including mannitol and hypertonic saline (HS) that are widely used to relieve cerebral oedema [3]. It is also well known that HS solutions may be more effective in ameliorating cerebral oedema than mannitol which is another classic osmotic dehydrating agent [3, 4]. The group of Schwab has been reported that 10 % HS not only effective in reducing the common cerebral oedema, but also for cases that failed with mannitol treatment [5].

The traditional theory that HS removes free water from the intracellular into the extracellular space through establishing osmotic pressure gradient and lowers peripheral vascular resistance has been accepted widely [6]. However, the action of HS beyond its osmotic effects has remained elusive. We have reported previously that HS could alleviate cerebral oedema through down-regulating the expression of aquaporin-4 in astrocytes in peri-ischemic hemispheric tissue [7, 8]. Moreover, it may be neuroprotective by reducing neuronal apoptosis [7] thus raising the possibility that HS alleviates cerebral oedema not only through an osmotic mechanism, but it may also mediate a non-osmotic molecular mechanism.

Cerebral oedema can be classified into three parts [9, 10], it starts from a cytotoxic brain oedema to ionic brain oedema, and then vasogenic brain oedema which is most severe with a widest range of injury. The degree of cerebral oedema is closely related to the change of BBB permeability. The BBB is composed of endothelial cells that line capillaries in brain parenchyma. Astrocytes, pericytes and perivascular microglia around capillaries are very important for maintaining the proper functioning of BBB [11, 12]. The up-regulation of the ionic channel and water transporter in these cells is pivotal to hyperpermeability in BBB [10, 13]. The above-mentioned had prompted us to hypothesize that water and/or ionic channel in cerebral cells is related to BBB permeability that may be the therapeutic targets of HS for ameliorating cerebral oedema.

VEGF is the major vascular permeability regulator on microvascular permeability. It has been reported that BBB permeability was significantly increased and the degree of cerebral oedema deteriorated abruptly after treatment with VEGF [14–16]. VEGFR2, a receptor of VEGF, plays an important role in regulating the VEGF function and increases vascular permeability as well [17, 18]. Many studies have reported that VEGF and VEGFR2 expression was significantly and time-dependently increased with increasing BBB permeability in acute phase of cerebral oedema caused by middle cerebral artery occlusion (MCAO) [16, 19, 20]. It has remained to be resolved whether VEGF and VEGFR2 expression would play a role in the therapeutic efficacy of HS in cerebral oedema.

Because HS can alleviate cerebral oedema by down-regulating aquaporin-4 as reported previously by us [6, 7], it was reasoned that it would also protect BBB permeability in the acute phase of cerebral oedema. The issue that follows is whether HS would regulate BBB permeability via regulating the expression of VEGF and VEGFR2 in astrocytes which are closely associated with the cerebral vessels.

In addition, the tight junction (TJ) between endothelial cells is the key element of BBB permeability, and disruption of tight junction leads to BBB breakdown [21]. Zonula occludens 1 (ZO-1), and claudin-5 are important components of tight junction, and the down-regulation of them would increase the permeability of BBB significantly [22]. It remains to be ascertained whether HS could regulate the expression of aforementioned tight junction proteins.

To ascertain these possibilities, the brain water content (BWC), Evan’s Blue (EB) extravasation, and the expression of VEGF, VEGFR2, ZO-1, claudin-5 mRNA and protein in peri-ischemic brain tissue were assessed in an MCAO model in rats. Furthermore, VEGF, VEGFR2 mRNA and protein expression in primary astrocytes was also examined. This aimed to determine if a functional correlation may be drawn among the various components both in vivo and in vitro.

## Methods

### Animals and experimental groups

207 SPF male Sprague-Dawley (SD) rats weighing 200–250 g (provided by Guangdong Medical Laboratory Animal Centre, Guangdong Province), are randomly divided into a sham-operated group (n = 69), cerebral ischemia + normal saline group (ischemia group, n = 69) and cerebral ischemic + 10 % HS treatment group (10 % HS group, n = 69). In ischemia group and 10 % HS group, rats were subjected to right-sided middle cerebral artery occlusion (MCAO). Reperfusion started at 2 h after MCAO; meanwhile, the rats in the ischemia group and 10 % HS group were treated with a continuous intravenous (i.v.) infusion of normal saline (0.3 ml/h) and 10 % HS, respectively. However, rats in sham-operated group were subjected to the same operating procedure but not subjected to MCAO. After this, the rats were treated with an i.v. infusion of normal saline (0.3 ml/h) via the tail vein until the end of the experiment. The rats in each group were further subdivided into three groups at various time points: 6, 12 and 24 h subgroups. Number of rats killed at various time points in different groups is shown in Table 1. All the animal experimental procedures were approved by Institutional Animal Care and Use Committee, Guangdong Province, China. All experiments were carried out in accordance with the National Institute of Health Guide for the Care and Use of Laboratory Animals.

**Table 1:** Number of rats killed at various time points in different groups

	BWC	Evans blue	RT-PCR/western blot	Double immunofluorescence
Sham group				
6 h	6	6	9	0
12 h	6	6	9	0
24 h	6	6	9	6
Ischemia group				
6 h	6	6	9	0
12 h	6	6	9	0
24 h	6	6	9	6
10 % HS group				
6 h	6	6	9	0
12 h	6	6	9	0
24 h	6	6	9	6

### Focal brain ischemia animal model

The rats were allowed free access to water but were not fed the night before prior to surgery. Anaesthesia was achieved with an intraperitoneal injection of 5 % Ketamine (40 mg/kg) before surgery. During the whole surgical procedure, a heating lamp was used to ensure that the rectal temperature was maintained between 37 and 37.5 °C. Focal cerebral ischemia was induced by intraluminal occlusion of the right middle cerebral artery as previously described [23, 24]. Briefly, after the exposure of the right common carotid artery (CCA), internal carotid artery (ICA), and external carotid artery (ECA) through a midline incision along the neck, a 4–0 head end spherical nylon suture was inserted into the right ICA to block the origin of right middle cerebral artery so that the blood flow to the cerebral somatosensory cortex was impeded. Sham-operated rats were subjected to the same surgical procedures without MCAO. At 2 h after MCAO, reperfusion was commenced by withdrawing the intraluminal filament which was followed by the neurologic assessments. The Longa scores test was used to evaluate neurologic deficits after MCAO [23]: 0, no observable neurologic deficit; (1) a mild focal neurologic deficit (failure to extend left forepaw fully); (2) a moderate focal neurologic deficit (circling to the left); and (3) a severe focal deficit (falling to the left); (4) no spontaneous motor activity (the rats did not walk spontaneously combined with depressed levels of consciousness). The rats with a score of 1–3 were used in experiments.

After decapitation, peri-ischemic brain tissues were obtained from the superior one-third of the cortical area, extending from longitudinal cerebral fissure to lateral cerebral fissure in the section, which was located at 7–11 mm posterior to the tip of olfactory bulb [25].

### Primary astrocyte cell culture and treatment

Rat primary astrocytes were isolated from the cerebrum of 0–24 h-old SD rats by a modification of a previously described method [26]. Briefly, rats were killed and their cerebral hemispheres were harvested. After the meninges and blood vessels were carefully cleared, the cortical tissues were collected into a beaker and dissected into tissue pieces of 1 mm^3^ size. This was followed by digesting in 0.125 % trypsin for 10 min at 37 °C. At the termination of digestion, the tissue pieces were manually dissociated by triturating with Dulbecco’s modified Eagle’s medium-F12 nutrient mixture (DMEM-F12) with 10 % fetal bovine serum. The cell suspension was then filtrated with a 70 μm cell strainer and centrifuged at 1500 rpm for 5 min. Finally, the supernatant was carefully discarded and the cells resuspended with 10 ml of DMEM-F12 supplemented with 10 % FBS. The cells were plated in 75 cm^2^ culture flasks at density of 2 × 10^6^ cells/ml and cultured at 37 °C in an incubator with 95 % air and 5 % CO_2_. When the culture reached its confluence, astrocytes were purified by shaking to remove most microglia and oligodendrocytes on an orbital shaker. The purified astrocytes were divided into three groups: control group, hypoxia + glucose free medium group (Hypoxia group) and hypoxia + glucose free medium +100 mM HS group (HS group). Hypoxia group and HS group were incubated at 37 °C incubator filled with 3 % oxygen and 5 % CO_2_ for 4 h, and the control group was cultured with DMEM-F12 containing 10 % FBS at 37 °C in humidified 5 % CO_2_ and 95 % air.

### Assessment of ischemic hemispheric brain water content

Brain oedema was estimated by comparing wet to-dry weight ratios [27]. Briefly, at the end of the experiment, rats were killed by decapitation under deep anesthesia. The brain was quickly removed and its moisture blotted gently. The brain was bisected through the inter-hemispheric fissure into right and left hemispheres. Subsequently, brain hemisphere was weighed on an electronic balance with a scale reading to within 0.001 mg. Dry weight of ischemic hemispheres was measured after the tissue was heated for 3 days at 100 °C in a drying oven. Tissue water content was then calculated as follows: % H_2_O = (wet weight − dry weight)/wet weight × 100 %.

### Evaluation of blood-brain barrier integrity

The integrity of the BBB was achieved by measuring Evans-Blue extravasated in ischemic hemispheric tissue as previously described [28, 29]. Evans Blue (EB, 2 % in saline, 4 ml/kg) was injected intravenously at the beginning of reperfusion. At 24 h after MCAO, the rats were transcardially perfused with 110 ml saline to remove the intravascular Evans blue dye under deep anesthesia. The brain was cut into 2 mm thick coronal sections at the level of the optic chiasma. The ischemic hemisphere was then weighed and homogenized in 2 ml 50 % trichloroacetic acid, followed by centrifugation at 10,000 rpm for 20 min. The supernatants were analysed at 620 nm using a spectrophotometer. The total Evans blue content in ischemic hemisphere was quantified in reference to a linear standard curve derived from known amounts of the dye and was expressed as micrograms per gram of tissue.

### RT real-time PCR

Total RNA was extracted from peri-ischemic brain tissue and primary astrocytes using the Trizol reagent according to the manufacturer’s protocol (Invitrogen Life Technologies Corporation, USA, Cat. No. 115596-026). Total RNA concentration was quantified with a spectrophotometer at 260 nm. Forward and reverse primer sequences for the respective genes are as follows: VEGF (93 bp), Forward: GCTTTACTGCTGTACCTCCAC, Reverse: AGAAGTTCGGCAGGACAC; VEGFR2 (180 bp), Forward: CAGACAGACAGTGGGATG GT, Reverse: GGTATCTGTGTCGTCTGAGTGA; β-actin (203 bp), Forward: GCCAACACAGTGCTGTCTG, Reverse: TACTCCTGCTTGCTGATCCA; ZO-1 (107 bp), Forward: GGAGCTACGCTTGCCACACT, Reverse: GGTCAATCAGGACAGAAACACA GT; Claudin-5 (302 bp), forward: TAAGGCACGGGTAGCACTCA, Reverse: GCCCAGCT CGTACTTCTGTG. The expression of target genes was measured in triplicate and normalized to β-actin, as an internal control.

Reverse transcription was carried out as follows: 500 ng RNA from the respective samples combined with 2 μl 5× PrimeScript^®^ Buffer (PrimeScript^®^ RT Master Mix, TaKaRa Biotechnology, Dalian, Co., Ltd, China; Cat No. DRR036S), RNase Free dH_2_O was added to 10 μl. After heated at 37 °C for 15 min and 85 °C for 5 s, the mixture was stored at −20 °C. For RT-PCR, the reaction mixture with a 10 μl final volume was composed of 5 μl 2 × SYBR Green I master mix, 1 μl cDNA, 0.5 μl of 10 μM forward primer, 0.5 μl of 10 μM reverse primer and 3 μl RNase Free dH_2_O. After the reaction mixture was pre-incubated at 95 °C for 30 s. The polymerase chain reactions (PCR) were performed according to the following procedures for 35 cycles: step 1: denaturation: 95 °C, 5 s; step 2: annealing: 60 °C, 30 s; step 3: elongation: 72 °C, 15 s. A modification of $2^{{\Delta \Delta - {\rm c}_{\text{t}} }}$ method was used to quantify the target gene expression [30].

### Western blot

Total protein was extracted from the peri-ischemic brain tissue and astrocyte culture using a protein extraction kit (KGP701, KeyGEN biotech, Nan Jing, China). Protein concentration of samples was quantified using BCA-100 protein quantitative analysing kit (KGP207, KeyGEN biotech, Nan Jing, China). Protein samples were separated by sodium dodecyl sulfate–polyacrylamide gels and transferred to polyvinylidene difluoride transfer membranes. The membranes were washed with TBS—0.1 % Tween buffer and blocked with 5 % non-fat dry milk for 1 h at room temperature, and incubated with primary antibody as follows: VEGF (1:1000, rabbit polyclonal IgG, Santa Cruz, USA, Cat. No. sc-152), VEGFR2 (1:1000; rabbit polyclonal IgG, Santa Cruz, USA, Cat. No. sc-505), ZO-1 (1:200; Rabbit polyclonal IgG; Invitrogen Life Technologies Corporation; Cat. No. 40-2200), Claudin-5 (1:500; Mouse monoclonal IgG; Invitrogen Life Technologies Corporation; Cat. No. 35-2500) and GAPDH (1:1000, Santa Cruz Biotechnology, USA, Cat. No. sc-20357) overnight at 4 °C. After washing in TBS-0.1 % Tween three times, they were incubated with the horseradish peroxidase (HRP)-conjugated secondary antibody (1:3500, goat anti-rabbit, Cell Signaling Technology, USA; Cat. No. 7074) for 1 h. The immunoblots were developed on Kodak films with an enhanced chemiluminescence detection system (Bei Jing Pu Li Lai Gene Technology Co., Ltd, China) according to the manufacturer’s instructions. The band intensity of VEGF and VEGFR2 expression levels relative to the GAPDH was quantified by FluorChem 8900 software (version 4.0.1, Alpha Innotech Corporation, USA).

### Double immunofluorescence

At 24 h after MCAO, coronal frozen sections of the brain of 30 μm thickness at the level of the optic chiasma were cut and rinsed in PBS. Sections were incubated in a humidified chamber with a mixture of glial fibrillary acidic protein (GFAP) (1:50, monoclonal antibody IgG, Millipore, USA, Cat. No. MAB 360) and VEGF (1:50, rabbit polyclonal IgG, Santa Cruz, USA, Cat. No. sc-152) or VEGFR2 (1:50; rabbit polyclonal IgG, Santa Cruz, USA, Cat. NO. sc-505) overnight at 4 °C. Subsequently, these sections were washed and incubated with Alexa Fluor^®^ 488 goat anti-rabbit IgG (H + L) (1:200; Invitrogen Life Technologies Corporation, USA; Cat. No. CA11008s) and Alexa Fluor^®^ 594 goat anti-mouse IgG (1:200; Invitrogen Life Technologies Corporation, USA; Cat. No. CA11005s) for 1 h at room temperature. After several washes with PBS, the sections were mounted with a fluorescent mounting medium. Colocalization was observed by confocal microscopy (Leica TCS SP2 AOBS; Leica Microsystems Ltd, Germany).

At 4 h after hypoxia, astrocytes in culture were fixed with 4 % paraformaldehyde for 20 min. Following rinsing with PBS, cells were blocked with 5 % goat serum for 30 min, and then incubated with a mixture of antibodies against GFAP (1:50; Millipore, USA, Cat. No. MAB 360) and VEGF (1:50; Santa Cruz, USA, Cat. No. sc-152) or VEGFR2 (1:50; Santa Cruz, USA, Cat. No. sc-505) at 4 °C overnight. On the following day, cells were incubated with Alexa Fluor^®^ 488 goat anti-rabbit IgG (H + L) (1:200; Invitrogen Life Technologies Corporation, USA; Cat. No. CA11008s) and Alexa Fluor^®^ 594 goat anti-mouse IgG (1:200; Invitrogen Life Technologies Corporation, USA; Cat. No. CA11005s) for 1 h at room temperature. After rinsing with PBS and mounted with a fluorescent mounting medium, the cells were examined under a fluorescence microscope (Olympus DP73 Microscope, Olympus, Tokyo, Japan).

### Statistical analysis

SPSS13.0 statistical software was used to analyze data. Different statistical methods were applied according to types of data. Values were expressed as mean ± standard deviation (±SD). Univariate-factor measurement data was analyzed by one-way ANOVA, but two-factor measurement data was analysed by two-way ANOVA. Multiple comparisons were analyzed by Fisher’s Least Significant Difference test method if the data was homogeneity of variance; otherwise, they were analyzed by Dunnett’s T3 method. The difference was considered statistically significance when *P* < 0.05.

## Results

### Ipsilateral ischemic hemispheric BWC

**Fig. 1:**
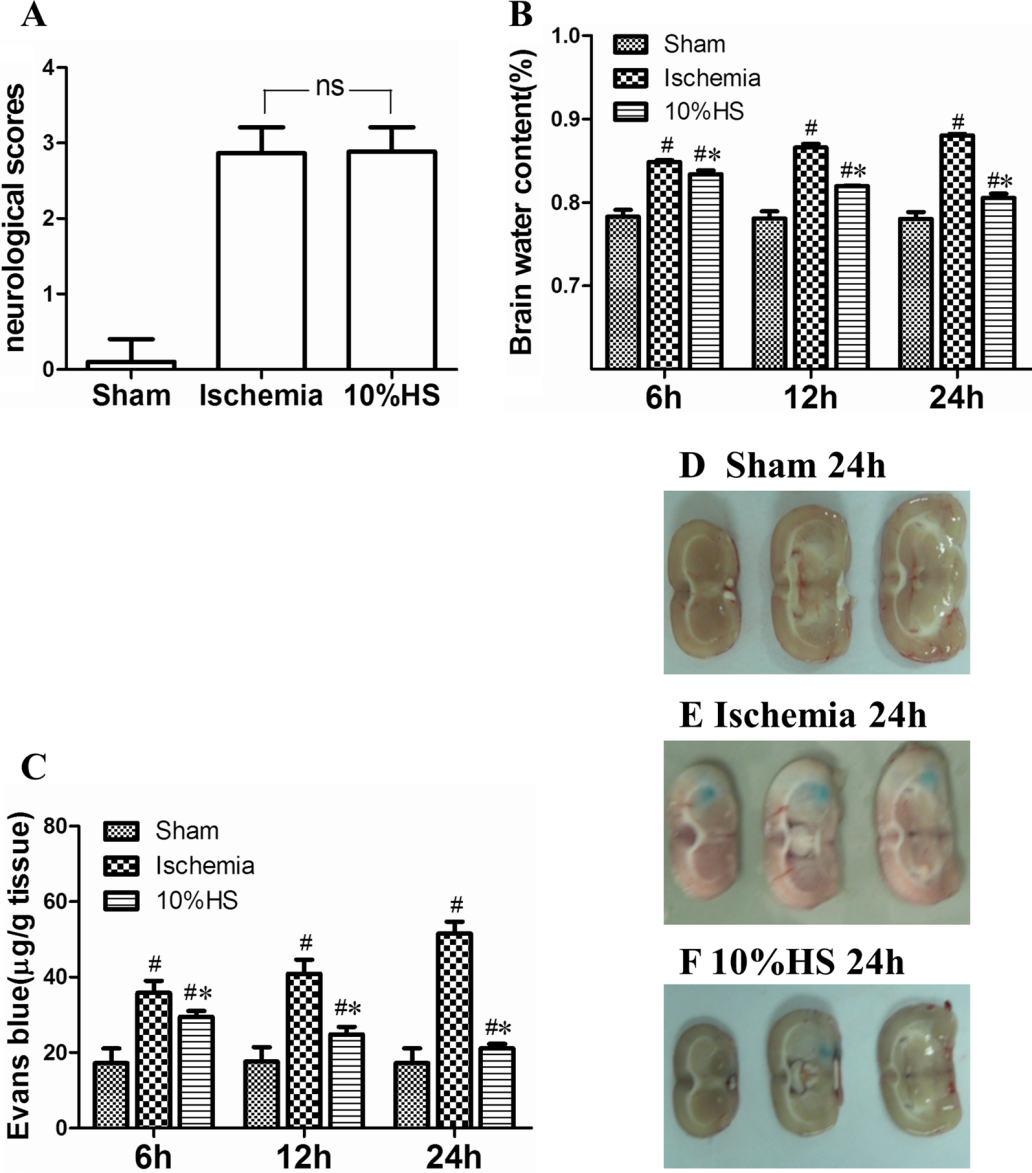
Assessment of BWC and Evans blue. *Bar graph* **A** the neurological score is not significantly different between ischemia group and 10 % HS group at 2 h after MCAO based on Zea-longa scores (ns: *P* > 0.05). *Bar graph* **B** the percentage of BWC in 10 % HS group was significantly decreased as compared with corresponding ischemia groups at 6, 12 and 24 h after MCAO (*P < 0.05). *Bar graph* **C** 10 % HS could reduce Evans blue extravasation effectively when compared with corresponding ischemia groups at 6, 12 and 24 h after MCAO (*P < 0.05). ^#^indicates compared with sham group, *P* < 0.05. **D**, **E** and **F** Evans blue extravasation in each group at 24 h after MCAO. The values are presented as the mean ± SD

The Longa scores showed that no significant difference between ischemia group and 10 % HS group (Fig. 1A; ns: *P* > 0.05). At 6, 12 and 24 h following MCAO, BWC in the ipsilateral ischemic hemispheres of ischemia group and 10 % HS group increased significantly when compared with that in the corresponding sham operated groups (Fig. 1B; ^#^ *P* < 0.05); but, the difference in the BWC between ischemia group and 10 % HS group at 6, 12 and 24 h following MCAO was significant (Fig. 1B; *P < 0.05). It is evident that BWC in ischemia group is significantly greater than that in 10 % HS group.

### Determination of BBB permeability with the use of Evans blue

The extravasation of EB was significantly increased in ischemia group and 10 % HS group at 6, 12 and 24 h as compared with the corresponding sham groups (Fig. 1C; ^#^ *P* < 0.05). However, after 10 % HS treatment, the concentration of EB in 10 % HS group was markedly decreased as compared with ischemia group (Fig. 1C; *P < 0.05). At 24 h after MCAO, EB staining area was significantly increased in brain sections of ischemia group, but was noticeably reduced with 10 % HS treatment (Fig. 1D–F).

### VEGF mRNA and protein expression in the peri-ischemic brain tissue

**Fig. 2:**
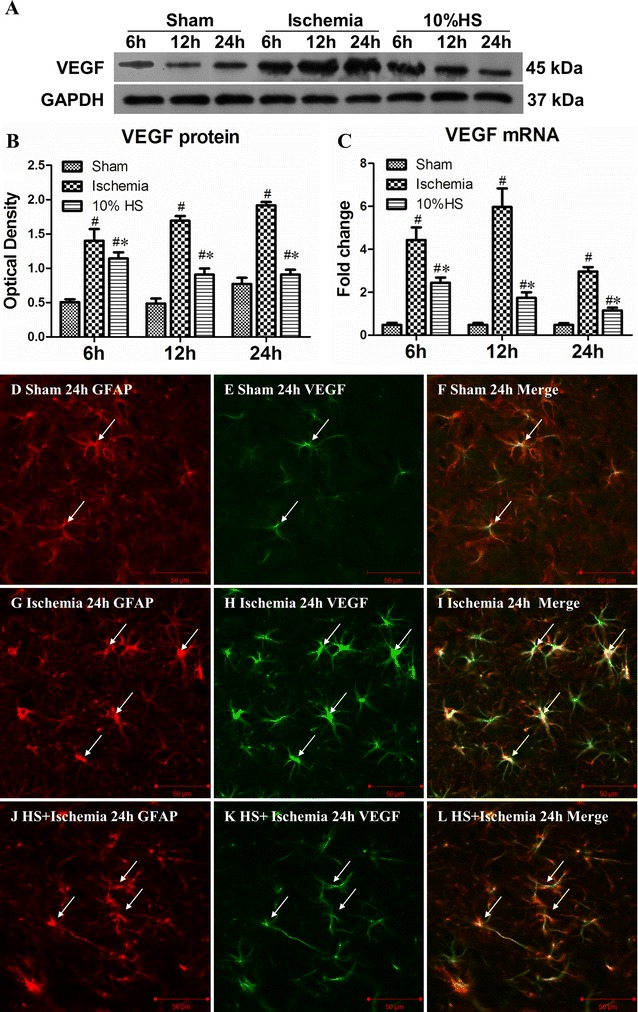
VEGF mRNA and protein expression in the peri-ischemic brain tissue in each group. **A** VEGF (45 kDa) and GAPDH (37 kDa) immunoreactive bands, respectively. *Bar graph* **B** depicts significant changes in the optical density of VEGF expression when compared with the corresponding ischemia groups (*P < 0.05). *Bar graph* **C** the fold change in VEGF mRNA expression. When compared with ischemia group at 6, 12 and 24 h after MCAO, VEGF mRNA expression in corresponding 10 % HS group is evidently reduced (*P < 0.05). ^#^indicates compared with sham group, *P* < 0.05. The values are presented as the mean ± SD. Confocal images showing the distribution of GFAP labeled astrocytes (**D**, **G**, **J**, *red*), VEGF (**E**, **H**, **K**, *green*), and GFAP labeling overlapping VEGF immunofluorescence can be seen in **F**, **I** and **L**. *Note* that VEGF expression in astrocytes (*arrows*) is markedly enhanced at 24 h following MCAO. However, after treatment with 10 % HS, it is noticeably reduced. *Scale bars* (**D**–**L**), 50 μm

Western blot showed a moderate expression of VEGF in the sham group. The expression level in the peri-ischemic brain tissue, however, was progressively increased up to 24 h; thus, at 6, 12 and 24 h after MCAO, compared with the corresponding sham groups, VEGF protein expression in hypoxia group was significantly increased (Fig. 2A, B; ^#^ *P* < 0.05). Remarkably, treatment with 10 % HS group markedly suppressed the VEGF protein expression when compared with the corresponding ischemia group (Fig. 2A, B; *P < 0.05).

RT-PCR showed that in ischemia group, VEGF mRNA expression in the peri-ischemic brain tissue was gradually increased at 6 and 12 h, followed by a decline at 24 h. At each time point after MCAO, VEGF mRNA expression was significantly up-regulated as compared with the corresponding sham group (Fig. 2C; ^#^ *P* < 0.05). As compared with the ischemia group, however, VEGF mRNA expression in 10 % HS group was significantly decreased (Fig. 2C; *P < 0.05).

In both sham and MCAO groups, VEGF expression was detected specifically in astrocytes, as confirmed by double immunofluorescence showing colocalization of GFAP. At 24 h after MCAO, VEGF immunoreactivity in the ischemia group (Fig. 2G–I) was markedly enhanced when compared with the sham group (Fig. 2D–F). In 10 % HS group (Fig. 2J–l), VEGF expression was evidently reversed as compared with the ischemia group. VEGF expression as detected either by western or immunofluorescence in 10 % HS group showed that despite its reduction, it remained above the basal levels as expressed by the sham group. Furthermore, a feature worthy of note is that astrocytes in hypoxia group appeared hypertrophic (Fig. 2G–I) when compared with that in 10 % HS group and sham group.

### VEGFR2 mRNA and protein expression in the peri-ischemic brain tissue

**Fig. 3:**
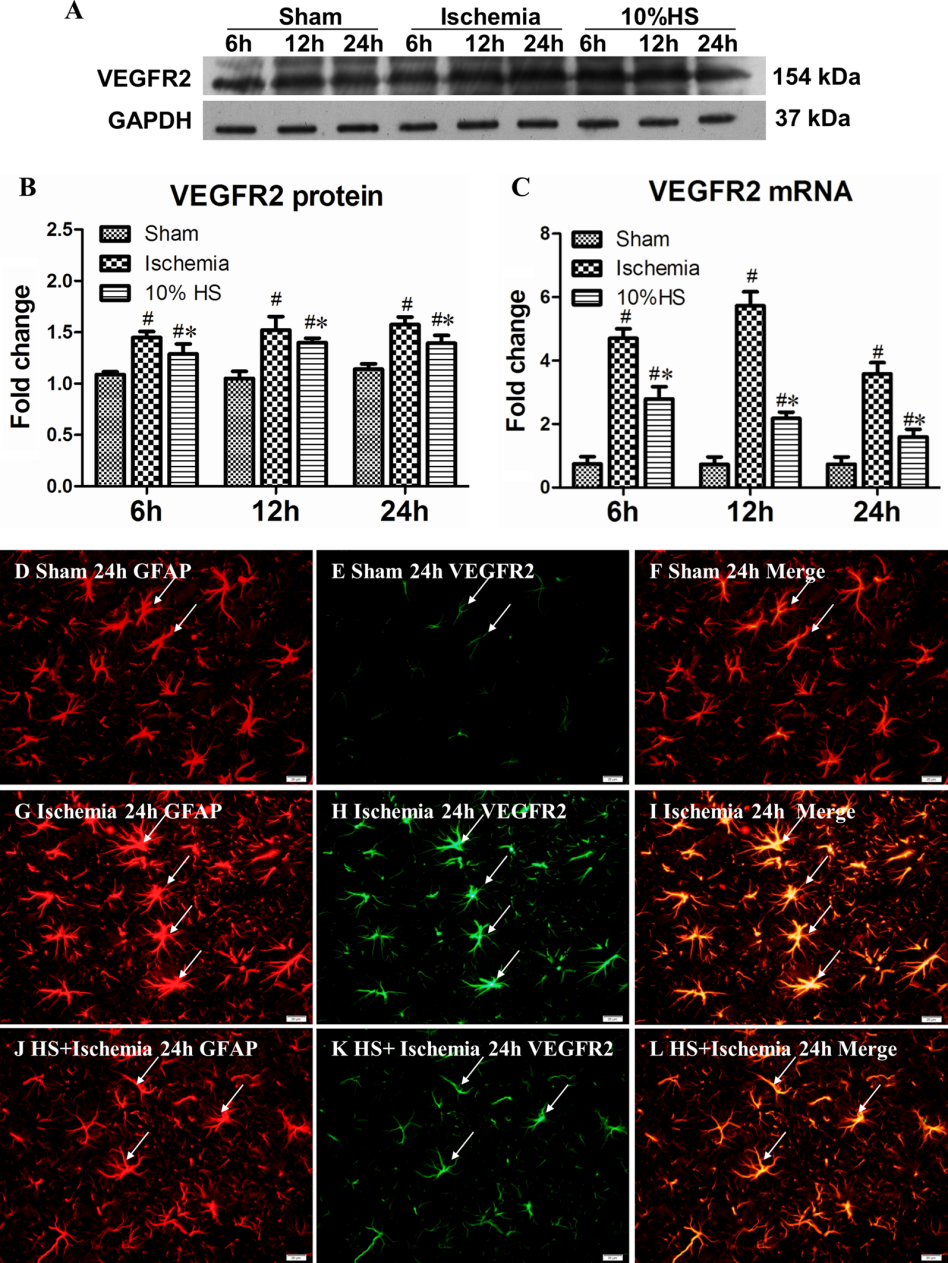
VEGFR2 mRNA and protein expression in the peri-ischemic brain tissue in each group. **A** VEGFR2 (154 kDa) and GAPDH (37 kDa) immunoreactive bands, respectively. *Bar graph* **B** the optical density of VEGFR2 expression in 10 % HS group at 6, 12 and 24 h after MCAO is significantly decreased when compared with the corresponding ischemia group (*P < 0.05). *Bar graph* **C** the fold change in VEGFR2 mRNA expression. Significant differences in mRNA level in ischemia group at 6, 12 and 24 h after MCAO is evident when compared with the corresponding 10 % HS groups (*P < 0.05). ^#^indicates compared with sham group, *P* < 0.05. The values are presented as the mean ± SD. Immunofluorescence images showing the distribution of GFAP (**D**, **G**, **J**, *red*) and VEGFR2 (**E**, **H**, **K**, *green*) in astrocytes (*arrows*) after MCAO for 12 h. Co-localization of GFAP and VEGFR2 can be seen in **F**, **I** and **L**. *Note* expression of VEGFR2 is down-regulated after treatment with 10 % HS. *Scale bars* (**D**–**L**), 20 μm

In ischemia group, an up-regulated VEGFR2 protein expression was observed in the peri-ischemic brain tissue at 6, 12 and 24 h after MCAO when compared with the corresponding sham group (Fig. 3A, B; ^#^ *P* < 0.05). After treatment with 10 % HS, however, VEGF protein expression in 10 % HS group was markedly decreased in comparison to the ischemia group (Fig. 3A, B; *P < 0.05).

A similar pattern change was observed in VEGFR2 mRNA whose expression was markedly increased in ischemia group at 6, 12 and 24 h following MCAO when compared with the corresponding sham group (Fig. 3C; ^#^ *P* < 0.05). Treatment with 10 % HS markedly suppressed the VEGFR2 mRNA expression level as compared with the corresponding ischemia group (Fig. 3C; *P < 0.05).

Double immunofluorescence labeling showed that VEGFR2 expression was specifically detected in cells, confirmed to be the astrocytes by double labeling with GFAP (Figs. 3D–L). At 24 h after MCAO, very intense VEGFR2 immunoreactivity was detected in ischemia group (Figs. 3G–I). It was also found in 10 % HS group (Figs. 3J–L) but the immunoreactivity was significantly decreased as compared with ischemia group.

### VEGF mRNA and protein expression in primary astrocytes

**Fig. 4:**
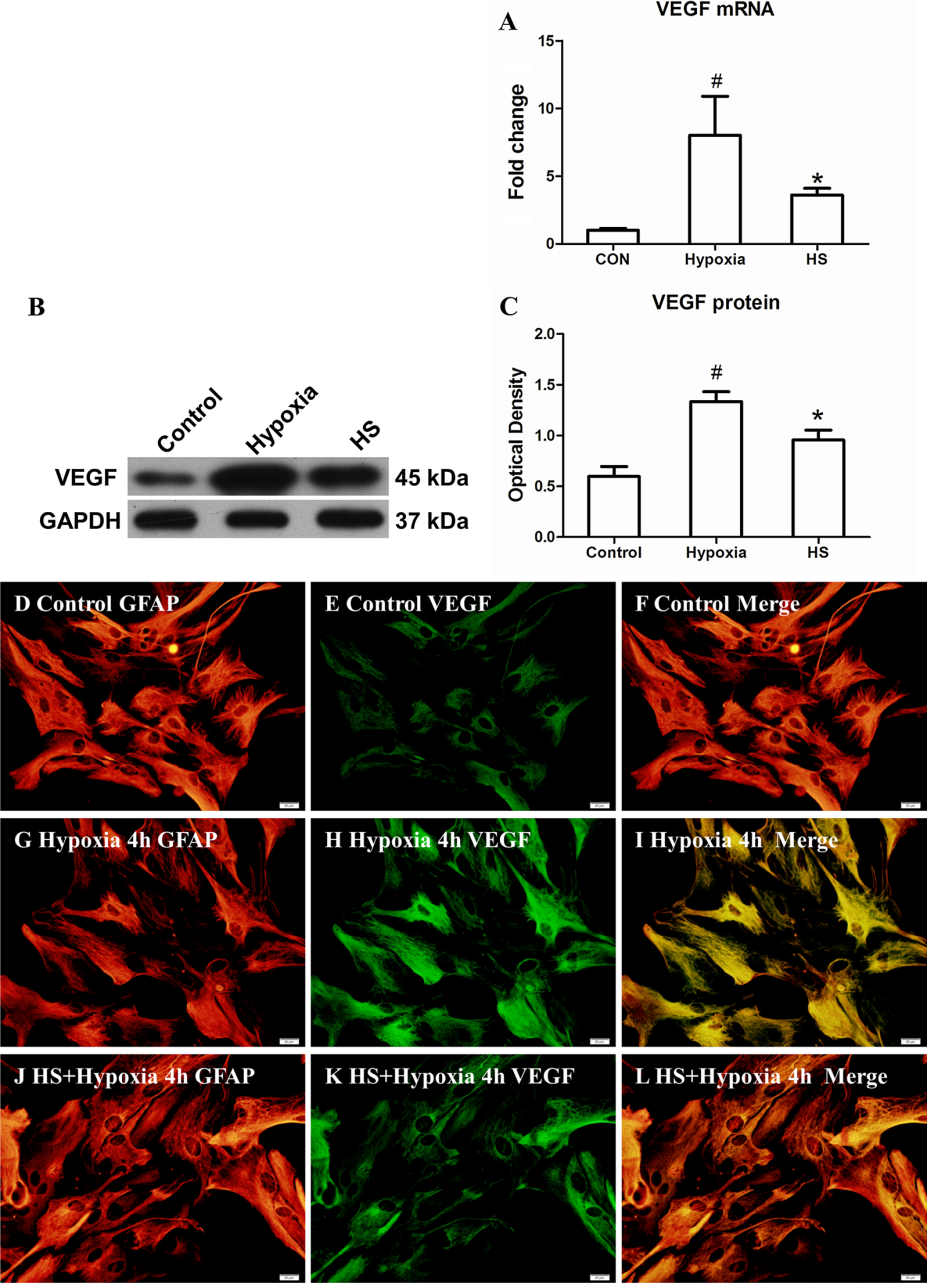
VEGF mRNA and protein expression in primary astrocytes. *Bar graph* **A** VEGF mRNA expression in HS group was significantly decreased in comparison to hypoxia group after hypoxia for 4 h. **B** VEGF (45 kDa) and GAPDH (37 kDa) immunoreactive bands, respectively. *Bar graph* **C** the optical density of VEGF expression in HS group was drastically attenuated when compared with the hypoxia group (*P < 0.05). ^#^indicates compared with sham group, *P* < 0.05. The values are presented as the mean ± SD. Immunofluorescence images showing GFAP labelled astrocytes (**D**, **G**, **J**, *red*), double labelled with VEGF (**E**, **H**, **K**, *green*). Co-localized expression of GFAP and VEGF in astrocytes can be seen in **F**, **I** and **L**. *Note* that VEGF expression in astrocytes is markedly enhanced at 24 h following MCAO. However, after treatment with 10 % HS, it is noticeably reduced. *Scale bars* (**D**–**L**), 20 μm

VEGF mRNA was significantly increased in primary astrocytes at 4 h after hypoxia in comparison with the control group (Fig. 4A; ^#^ *P* < 0.05), but after treatment with 100 mM HS, VEGF mRNA in primary astrocytes in HS group was significantly decreased when compared with that in hypoxia group (Fig. 4A; *P < 0.05). The immunoreactive band of VEGF protein levels that appeared at approximately 45 kDa, increased significantly in primary astrocytes at 4 h after hypoxia when compared with that in control group (Fig. 4B, C; ^#^ *P* < 0.05). However, the optical density of VEGF protein in HS group that was treated with 100 mM HS was significantly decreased (Fig. 4B, C; *P < 0.05).

The results of double immunofluorescence showed that VEGF expression in astrocytes was completely co-localized with GFAP labeling. Very weak VEGF immunoreactivity was detected in control group (Fig. 4D–F). At 4 h after hypoxic exposure, VEGF immunoreactivity was markedly enhanced in astrocytes (Fig. 4G–L) as compared with the control group (Fig. 4D–F), but VEGF immunoexpression was evidently decreased at 4 h after treatment with 100 mM HS (Fig. 4J–L) and was comparable to that in the hypoxia group (Fig. 4G–I).

### VEGFR2 mRNA and protein expression in primary astrocytes

**Fig. 5:**
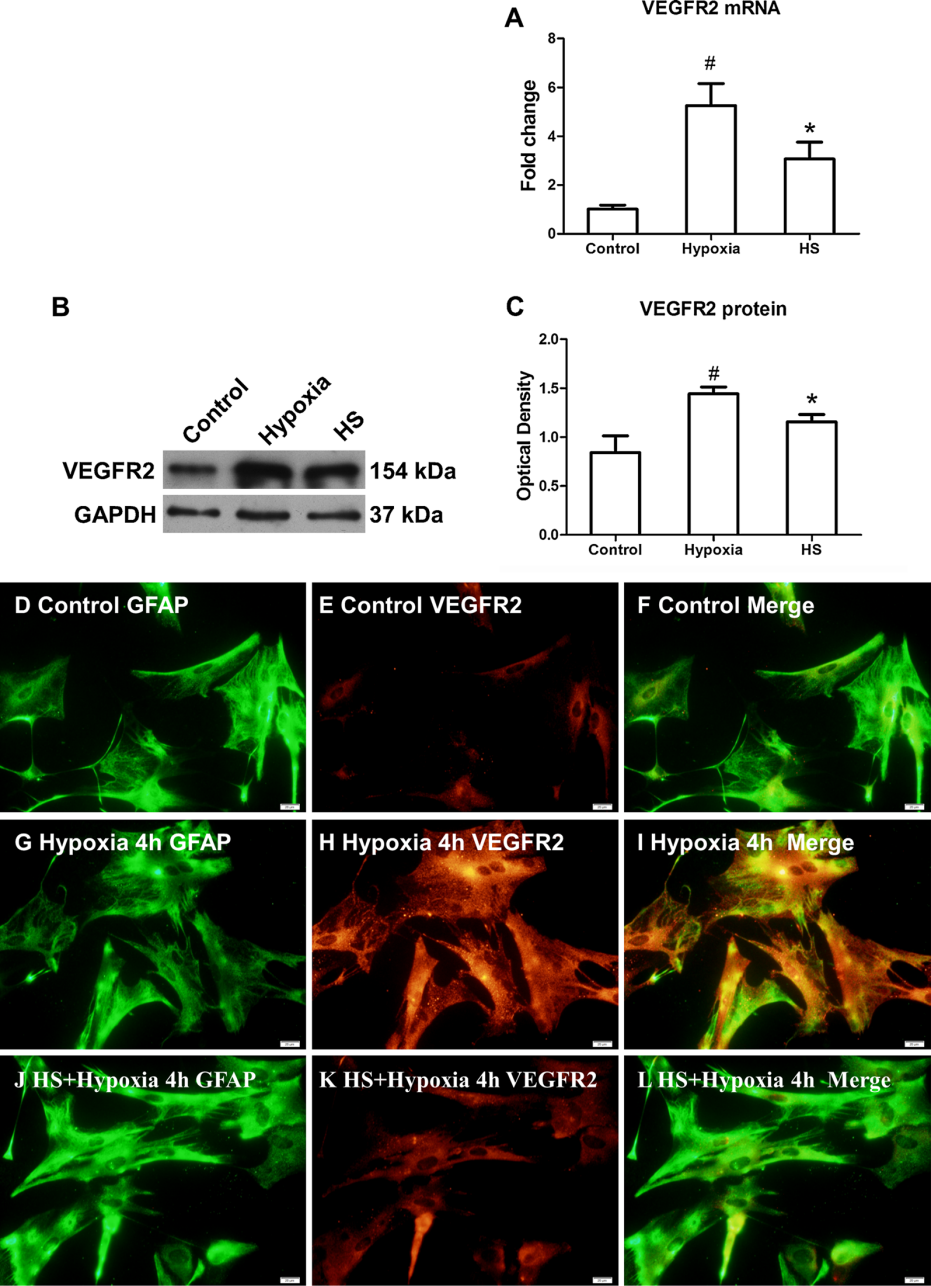
VEGFR2 mRNA and protein expression in primary astrocytes. *Bar graph* **A** VEGFR2 mRNA expression in HS group was significantly decreased in comparison to hypoxia group after hypoxia for 4 h. **B** VEGFR2 (154 kDa) and GAPDH (37 kDa) immunoreactive bands, respectively. *Bar graph* **C** the optical density of VEGFR2 expression in HS group was drastically attenuated when compared with the hypoxia group (*P < 0.05). ^#^indicates compared with sham group, *P* < 0.05. The values are presented as the mean ± SD. Double immunofluorescence images show that the expression of GFAP (**D**, **G**, **J**, *red*), VEGFR2 (**E**, **H**, **K**, *green*). The co-localized expression of GFAP and VEGFR2 can be seen in **F**, **I** and **L**. *Note* expression of VEGFR2 is markedly enhanced after hypoxia which is reduced by HS. *Scale bars* (**D**–**L**), 20 μm

At 4 h after hypoxia, VEGFR2 mRNA was significantly increased in primary astrocytes when compared with the control group (Fig. 5A; ^#^ *P* < 0.05), but following treatment with 100 mM HS, VEGFR2 mRNA in primary astrocytes in HS group was significantly decreased in comparison with that in hypoxia group (Fig. 5A; *P < 0.05). Western blot analysis showed that VEGFR2 protein levels were significantly increased in primary astrocytes under hypoxic condition for 4 h when compared with that in control group (Fig. 5B, c; ^#^ *P* < 0.05). But it was decreased significantly in HS group that was treated with 100 mM HS (Fig. 5B, C; *P < 0.05).

Double immunofluorescence labeling showed that the GFAP labeling in astrocytes was totally coincident with VEGFR2 expression (Fig. 5D–L). Following hypoxic exposure for 4 h, VEGFR2 immunofluorescence in astrocytes was markedly increased in hypoxia group (Fig. 5G–I). However, it was noticeably attenuated in 10 % HS group (Fig. 5J–L) when compared with hypoxia group.

### Zo-1, claudin-5 mRNA and protein expression in the peri-ischemic brain tissue

**Fig. 6:**
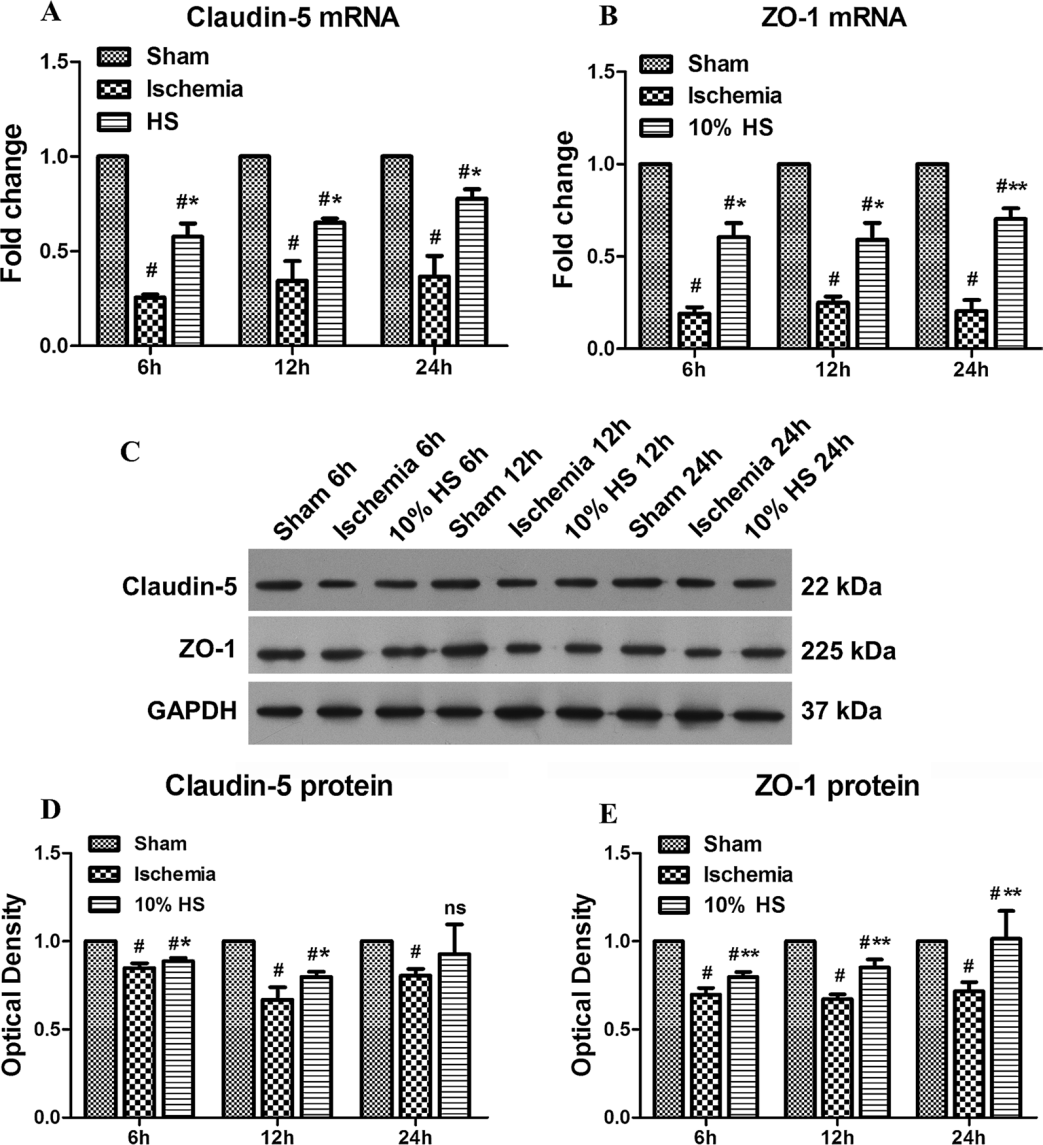
Zo-1, claudin-5 mRNA and protein expression in the peri-ischemic brain tissue in each group. *Bar graphs* **A**, **B** the mRNA expressions of Zo-1, claudin-5 were significantly decreased at 6, 12 and 24 h after MCAO, respectively, as compared with the corresponding sham group (^#^indicates compared with the corresponding sham group, *P* < 0.05), but they were increased significantly at corresponding ischemia group after treatment with 10 % HS (*P < 0.05, **P < 0.01). **C** Zo-1 (225 kDa), claudin-5 (22 kDa) and GAPDH (37 kDa) immunoreactive bands, respectively. As seen in *Bar graphs* **D**, **E** the optical density of Zo-1, claudin-5 was significantly attenuated after MCAO at 6, 12 and 24 h (^#^indicates compared with sham group, *P* < 0.05). But 10 % HS could inhibit the down-regulation of Zo-1, claudin-5 protein expression effectively at 6, 12 h after MCAO (*P < 0.05, **P < 0.01, *ns* non-significant). The values are presented as the mean ± SD

Zo-1 and claudin-5 mRNA expression level was significantly decreased at 6, 12 and 24 h following MCAO (Fig. 6A, B; ^#^ *P* < 0.05); however, at 6, 12 and 24 h after 10 % HS treatment, Zo-1 and claudin-5 were increased significantly when compared with the corresponding ischemia group (Fig. 6A, B; *P < 0.05).

At 6, 12 and 24 h after MCAO, Zo-1 and claudin-5 proteins were significantly decreased (Fig. 6C, E; ^#^ *P* < 0.05), but 10 % HS inhibited the down-regulation of them at each time point (Fig. 6C, E; *P < 0.05, **P < 0.01).

## Discussion

This study has shown that HS could significantly decrease cerebral oedema. The present results suggest that such an effect may be through restoring the BBB integrity as evidenced by the reduced permeability to exogenous tracer EB that inundated the ischemic tissue after MCAO. Concomitant to this is reduced VEGF, VEGFR2 and tight junction proteins such as ZO-1 and claudin-5 expression suggesting their involvement in this process. It is therefore suggested that 10 % HS is BBB protective in ischemic injuries.

Like most osmotic agents, HS could improve arterial pressure, lower ICP, and increase cerebral blood flow and oxygen delivery. It has been widely used for the treatment of cerebral oedema because of its efficacy of decreasing BWC [3]. In the present ischemia–reperfusion injury experimental model, an obvious cerebral oedema was observed; but, after treatment with 10 % HS, the BWC of 10 % HS group was significantly decreased as compared with the corresponding ischemia group. These results are consistent with the previous studies that HS could drastically reduce BWC [31–33] and, hence, cerebral oedema.

It has been reported that the alteration of BBB permeability and the damage of BBB integrity were closely related to cerebral oedema [34]. BBB is a selective semi-permeability membrane. It separates the brain parenchyma from the peripheral circulation and stabilizes the microenvironment of neurons, keeping it from deleterious effects of certain substances from blood [11, 35]. The disruption of BBB will lead to extravasation of intravascular substances into the brain extracellular spaces. The present results have shown that EB extravasation in the ischemic hemisphere was gradually increased up to 24 h. This was coupled by an increase in BWC after ischemia–reperfusion. The results are therefore in accord with previous report that ischemia–reperfusion caused an up-regulation of BBB permeability and breakdown [28]. As expected, after treatment with 10 % HS, EB extravasation was significantly decreased. These results demonstrate that 10 % HS ameliorates cerebral oedema through reduction of BBB permeability and protection of BBB integrity. The pertinent question arose from this was whether HS could perform such a function.

VEGF is known as a pivotal vascular permeability factor. In some pathological situations, such as MCAO [36], and brain injury [37], VEGF was remarkably increased in the border area of the lesion. Increased VEGF has been reported to induce leakage of BBB [38, 39]. This not only leads to the formation of cerebral oedema but also aggravates it [38, 40, 41]. Application of a neutralizing anti-VEGF antibody can reverse the cerebral oedema caused by VEGF [39]. It has also been reported that rats treatment with VEGF protein in early phase of cerebral ischemic leads to increase in BBB permeability, or even intracerebral haemorrhage which are reduced by VEGF inhibitor [38].

VEGFR2, a major receptor of VEGF, is also an important mediator of vascular permeability [42]. It is overexpressed in endothelial cells, astrocytes, neuronal somata and processes adjacent to the damage after brain injury [42]. Previous reports have suggested that VEGFR2, like VEGF, increases with increasing BBB permeability during the acute phase of cerebral oedema induced by various injuries [16, 19, 43]. Moreover, the effects of VEGF on BBB junction components were VEGFR2 dependent, and inhibition of VEGF–VEGFR2 axis is beneficial to ameliorate oedema [44].

The above-mentioned findings have demonstrated that BBB permeability and integrity are closely linked to VEGF and VEGFR2. As a corollary, targeting at VEGF and VEGFR2 may prove to be a potential therapeutic strategy to prevent brain oedema formation. In view of this, we further defined whether HS would affect the expression of VEGF and VEGFR2. Indeed we show here that expression of VEGF, VEGFR2 mRNA and protein was significantly up-regulated at 6 h after MCAO peaking at 12 h. More strikingly, when compared with the ischemia group, the 10 % HS group displays a lower VEGF, VEGFR2 mRNA and protein expression. The changes correlate with the reduced BBB permeability after treatment with 10 % HS. This suggests that HS may down-regulate the BBB permeability through inhibiting VEGF and VEGFR2 expression. Hypoxia inducible factor-1 (HIF-1) has been implicated in the expression of VEGF [45, 46] and this may offer explanation for the inhibition of HS on VEGF, but further studies are necessary to confirm this possibility.

It is known that astrocytes play a significant role in maintaining BBB permeability and integrity [11, 47, 48]. In CNS inflammatory disease, astrocyte-derived VEGF-A could aggravate BBB disruption. Inhibition of the VEGF expression in astrocytes plays a protective role in BBB function [47]. The present results have shown that the VEGF expression in astrocytes was remarkably increased after MCAO, and 10 % HS could down-regulate it effectively. This suggests that VEGF expression is increased in astrocytes after ischemia–reperfusion and HS intervention could inhibit its expression.

In vitro, hypoxia model of primary astrocytes was also performed to further determine the effect of HS on the expression of VEGF and VEGFR2 in astrocytes. The results are in concert with in vivo experiments. The levels of VEGF, VEGFR2 mRNA and protein expression were significantly augmented, but they were markedly depressed after treatment with 100 mM HS. Double immunofluorescence labelling also supported this which demonstrates that HS could directly suppress the expression of VEGF and VEGFR2 in astrocytes.

Finally, it remains to be explained on why inhibition of HS on VEGF and VEGFR2 expression had a beneficial effect on down-regulating the permeability of BBB. The reason for this may be that VEGF could regulate the expression of components of the tight junction such as ZO-1, claudin-5 etc.

ZO-1 and claudin-5 are the main components of tight junction. Previous study has showed that VEGF could down-regulate ZO-1, and claudin-5 expression through PLCγ/PKC/eNOS signaling pathway leading to the disruption of BBB. Silencing of VEGF expression in astrocytes by using siRNA, or inhibiting PLCγ/PKC/eNOS signaling pathway in endothelial cells by specific inhibitor of PLCγ and eNOS could inhibit down-regulation of claudin-5 and hence, the permeability of BBB is reduced [47].

In this study, HS could also inhibit the down-regulation of ZO-1, and claudin-5 significantly. Therefore, the mechanism whereby HS could ameliorate cerebral oedema may be via inhibition of VEGF-mediated down-regulation of ZO-1, and claudin-5 to protect the integrity of BBB. It is suggested that the PLCγ/PKC/eNOS signaling pathway maybe involved in this process, but it needs to be further clarified.

It has been reported that VEGF expression is also detected in vascular endothelial cells and neurons. The possibility that HS can also inhibit VEGF expression in these cells should therefore be considered. In addition, VEGF is also known as an important driver of immune cell infiltration [49–51]. This implicates that inhibition of VEGF may be beneficial to reduce inflammatory response as well. We had reported previously that HS could significantly decrease TNF-α and IL-1β expression levels through inhibition of JNK signaling pathway in microglia [52]. It is well documented that inflammatory cytokines such as TNF-α and IL-1β are also produced by astrocytes via JNK signalling pathway [53], and that they are known to be mediators of BBB disruption [12]. Therefore, decreasing the production of proinflammatory cytokines released by astrocytes by HS may prove to be beneficial or protective for the permeability integrity of BBB. In the lack of experimental evidence for this in this study, this remains purely speculative. This study showed that the augmented VEGF and VEGFR2 expression after ischemia–reperfusion plays an important role in increasing BBB permeability that exacerbates cerebral oedema as manifested by the leakage of EB in the peri-ischemic brain tissue and the increase of BWC. HS therefore has additional role by protecting BBB permeability and can ameliorate cerebral oedema through inhibition of VEGF and VEGFR2-mediated tight junction disruption.

## Conclusion

In conclusion, we have shown that ischemia–reperfusion induced up-regulated VEGF and VEGR2 expression on astrocytes leading to subsequent down-regulation of ZO-1 and claudin-5 which contributes to BBB dysfunction notably its increased permeability but that may be effectively reduced by HS. This has furthered and amplified our understanding of the mechanistic and molecular roles of HS in clinical management of cerebral oedema.

## Abbreviations

HS: hypertonic saline
BWC: brain water content
VEGF: vascular endothelial growth factor
VEGFR2: vascular endothelial growth factor receptor 2
EB: Evans blue
MCAO: middle cerebral artery occlusion
CCA: common carotid artery
ECA: external carotid artery
ICA: internal carotid artery
DMEM-F12: Dulbecco’s Modified Eagle Medium/Nutrient Mixture-F12
FBS: fetal bovine serum
GFAP: glial fibrillary acidic protein
BBB: blood–brain barrier
